# Inducibility of ventricular fibrillation during mild therapeutic hypothermia: electrophysiological study in a swine model

**DOI:** 10.1186/s12967-015-0429-9

**Published:** 2015-02-22

**Authors:** Jaroslav Kudlicka, Mikulas Mlcek, Jan Belohlavek, Pavel Hala, Stanislav Lacko, David Janak, Stepan Havranek, Jan Malik, Tomas Janota, Petr Ostadal, Petr Neuzil, Otomar Kittnar

**Affiliations:** Department of Physiology, First Faculty of Medicine, Charles University in Prague, Albertov 5, Prague 2, 128 00 Czech Republic; 3rd Department of Medicine, First Faculty of Medicine, Charles University in Prague and General University Hospital, U Nemocnice 2, Prague 2, 128 00 Czech Republic; 2nd Department of Medicine, First Faculty of Medicine, Charles University in Prague and General University Hospital, U Nemocnice 2, Prague 2, 128 00 Czech Republic; Department of Cardiology, Na Homolce Hospital, Roentgenova 2/37, Prague 5, 150 30 Czech Republic; 2nd Department of Surgery, Cardiovascular Surgery, First Faculty of Medicine, Charles University in Prague and General University Hospital, U Nemocnice 2, Prague 2, 128 00 Czech Republic

**Keywords:** Mild therapeutic hypothermia, Ventricular fibrillation, Hypokalemia, Long QT interval

## Abstract

**Introduction:**

Mild therapeutic hypothermia (MTH) is being used after cardiac arrest for its expected improvement in neurological outcome. Safety of MTH concerning inducibility of malignant arrhythmias has not been satisfactorily demonstrated. This study compares inducibility of ventricular fibrillation (VF) before and after induction of MTH in a whole body swine model and evaluates possible interaction with changing potassium plasma levels.

**Methods:**

The extracorporeal cooling was introduced in fully anesthetized swine (n = 6) to provide MTH. Inducibility of VF was studied by programmed ventricular stimulation three times in each animal under the following: during normothermia (NT), after reaching the core temperature of 32°C (HT) and after another 60 minutes of stable hypothermia (HT60). Inducibility of VF, effective refractory period of the ventricles (ERP), QTc interval and potassium plasma levels were measured.

**Results:**

Starting at normothermia of 38.7 (IQR 38.2; 39.8)°C, HT was achieved within 54 (39; 59) minutes and the core temperature was further maintained constant. Overall, the inducibility of VF was 100% (18/18 attempts) at NT, 83% (15/18) after reaching HT (*P* = 0.23) and 39% (7/18) at HT60 (*P* = 0.0001) using the same protocol. Similarly, ERP prolonged from 140 (130; 150) ms at NT to 206 (190; 220) ms when reaching HT (*P* < 0.001) and remained 206 (193; 220) ms at HT60. QTc interval was inversely proportional to the core temperature and extended from 376 (362; 395) at NT to 570 (545; 599) ms at HT. Potassium plasma level changed spontaneously: decreased during cooling from 4.1 (3.9; 4.8) to 3.7 (3.4; 4.1) mmol/L at HT (*P* < 0.01), then began to increase and returned to baseline level at HT60 (4.6 (4.4; 5.0) mmol/L, *P* = NS).

**Conclusions:**

According to our swine model, MTH does not increase the risk of VF induction by ventricular pacing in healthy hearts. Moreover, when combined with normokalemia, MTH exerts an antiarrhythmic effect despite prolonged QTc interval.

## Introduction

Mild therapeutic hypothermia (MTH) is recommended in patients remaining comatose after cardiac arrest caused by ventricular fibrillation (VF) [1-5] and should be considered in those with other presenting rhythms [6-8]. According to animal studies [9-13], early induction of MTH is supposed to be beneficial, but clinical trials did not prove it consistently yet [14-17]. Consequently, earliest possible induction of MTH is emphasized in some recent protocols using nasopharyngeal evaporative cooling and peripheral veno-arterial extracorporeal membrane oxygenation (ECMO) hoping to improve the neurological outcome [18-22]. On the other hand, the occurrence of malignant ventricular tachyarrhythmias during MTH still remains a major cause of death considering predisposing factors such as myocardial ischemia/reperfusion damage, often reported hypokalemia during the cooling phase [23-25], slowing of the heart rate and QT interval prolongation [25-29].

The aim of this experimental study was to assess the safety of MTH in terms of inducibility of malignant ventricular tachyarrhythmias in relation to spontaneous changes of potassium plasma level and QT interval in a whole body pig model. We hypothesized that MTH related hypokalemia together with prolongation of the QT interval might predispose the heart to greater electrical instability and lower VF threshold.

## Methods

The experimental protocol was designed to perform electrophysiological (EP) study before and after the induction of MTH using ECMO to provide cooling and hemodynamic support during the stimulation protocol and arrhythmias. All experiments were performed on cross-bred swine (Landrace x White) in an accredited university experimental laboratory by the skilled team of intensive care specialists and laboratory and veterinary technicians. The animals were handled in accordance with the guidelines of research animal use [30]. The protocol was approved by the Charles University First Faculty of Medicine Institutional Animal Care and Use Committee and performed at the Animal Laboratory, Department of Physiology, First Faculty of Medicine, Charles University in Prague in accordance with Act No 246/1992 as amended, Collection of Laws, Czech Republic, that is harmonized with EU Directives 86/609/EEC as amended, 2007/526/ES, 2010/63/EU.

### Anesthesia and monitoring

**Figure 1:**
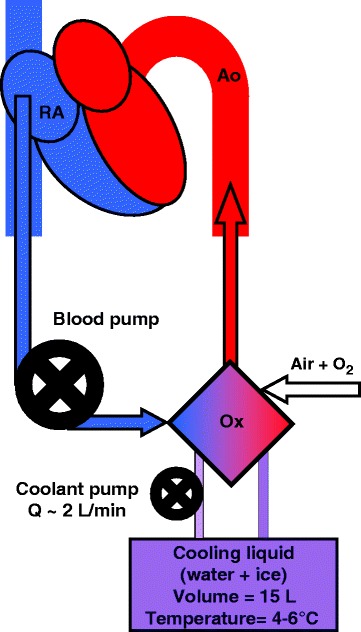
**V-A ECMO circuit.** Blood is pumped from the right atrium via oxygenator where blood is cooled and gases are exchanged into abdominal aorta. The custom made cooling circuit was assembled from the pump and reservoir filled with refrigerated water and melting ice. RA indicates right atrium; Ox, oxygenator; Ao, abdominal aorta.

After intramuscular sedation and premedication (azaperone 2–3 mg/kg, ketamine 20 mg/kg all i.m.) the marginal ear vein was cannulated and the pigs were preoxygenated with 100% oxygen via a facial mask. General anesthesia was induced by intravenous bolus of propofol (1–2 mg/kg) and orotracheal intubation was performed. Mechanical ventilation was adjusted by Intellivent-ASV closed-loop system (G5, Hamilton Medical, Bondauz, Switzerland) to maintain normoxia (SpO_2_ > 97%, pO_2_ 100 mmHg) and normocapnia (EtCO_2_ 38–40 mmHg) respecting the actual metabolic rate. The total intravenous anesthesia was maintained by continuous administration of propofol (6–12 mg/kg/h) and morphine (0.1-0.2 mg/kg/h), pipecuronium boluse (4 mg i.v.) were applied when shivering occurred during MTH. The depth of anesthesia was regularly assessed by the photoreaction and the corneal reflex and adjusted accordingly. Intravenous infusion of Ringer’s solution was given to reach and maintain central venous pressure between 6 and 8 mmHg. Anticoagulation was provided by unfractionated heparin bolus (100 IU/kg i.v.) followed by continuous intravenous drip (40–50 IU/kg/h) to maintain target activated clotting time 180–250 seconds (values checked every hour with Hemochron Junior+, International Technidyne Corporation, USA). Sheaths and catheters were inserted to femoral and carotid/jugular vessels as needed. Invasive blood pressure from carotid and pulmonary artery, central venous pressure (TruWave, Edwards Lifesciences, USA), body surface ECG, capnometry and pulse oximetry were continuously monitored by bedside monitor (Life Scope TR, Nihon Kohden, Japan) and trends were recorded. Mixed venous oximetry (SvO_2_), continuous cardiac output and pulmonary artery temperature were recorded using Swan-Ganz Combo V cathether (Vigilance monitor, Edwards Lifesciences, USA). A diagnostic decapolar catheter (Response CSL, St. Jude Medical, USA) was inserted under fluoroscopic guidance into the apex of the right ventricle (RV) via jugular vein to provide monitoring of intracardial electrograms and to induce VF. Five bipolar channels were recorded from the apex to base of RV at 3 kHz sampling rate. Heart rate and QT interval were manually measured and QTc was calculated using Bazett’s formula (QT interval divided by square root of RR interval). Before starting the protocol, cardiopulmonary bypass was established (Figure 1): an inlet 19 F cannula was inserted via femoral vein into the right atrium and an outlet 17 F cannula into abdominal aorta via femoral artery. The cannulae were connected to ECMO circuit consisting of a blood pump (Levitronix Centrimag, Levitronix, USA) and oxygenator (Quadrox, Maquet, Germany). A custom made cooling system was connected to the oxygenator. The cooling system consisted of a rotary pump and reservoir (volume of 15 L) filled with refrigerated water with ice cubes, which were gradually supplemented to keep the temperature of the coolant between 4 and 6°C. The coolant flow rate was set up to approximately 2 L/min during the cooling phase a consequently was slowed as needed to maintain the core temperature of 32°C. The ECMO circuit was primed with 500 mL of normal saline with 2500 IU of unfractionated heparin. The ECMO blood flow was empirically set at 40 mL/kg/min to provide the cooling and to prevent low cardiac output and hypotension during the stimulation protocol. After the onset of VF, the flow was increased to 80–100 mL/kg/min as needed to keep mean arterial pressure of 60 mmHg until an effective pulsatile sinus rhythm with mean arterial pressure above 60 mmHg was restored. Blood gasses, pH and potassium plasma levels were continuously sampled and recorded in ECMO circuit by a real-time analyzer (CDI 500, Terumo, Japan). At baseline and during the experiment was the real-time analyzer calibrated in 15 minute intervals using a bedside blood analysis system (IRMA TruPoint, International Technidyne Corporation, USA). Despite the expected hypokalemia during MTH, no potassium was replaced during the whole protocol, therefore all changes of potassium plasma levels were spontaneous. Ventilation support, as well as ECMO gas flow, was regularly adjusted to reach the target values (pH 7.4, pO_2_ 100 mmHg and pCO_2_ 38-40 mmHg).

### Electrophysiological study

**Figure 2:**
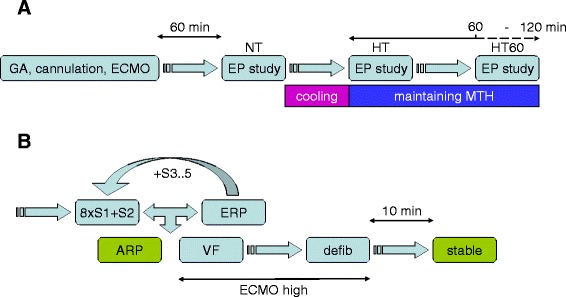
**Diagram of study protocol (A) and electrophysiological study (B).** GA indicates induction of general anesthesia; ECMO, initiation of ECMO; EP study, electrophysiological study; HT, hypothermia (core temperature of 32°C); 8 × S1, train of eight basic stimuli; S2-5, up to five extrastimuli; ERP, effective refractory period; ARP, absolute refractory period; VF, ventricular fibrillation; defib, defibrillation; steady, steady state; ECMO high, increasing of the ECMO flow to 80-100 mL/kg/min after onset of VF until the sinus rhythm was restored.

VF was induced by programmed ventricular stimulation (PVS, Figure 2B). Briefly, electrical stimuli (12 ms duration, maximal stimulation current 20 mA) were delivered from the apex of RV. Eight basic stimuli (S1) of cycle length 350 or 400 ms according to the heart rate were coupled with up to 4 extrastimuli (S2-S5). The coupling interval of S2 was decreased at 10 ms steps until the ventricular effective refractory period (ERP) was achieved. S2 coupling interval was then set to 10 ms above ERP and analogically S3 or S4 stimuli were delivered until VF or absolute ventricular refractory period (ARP) was reached. There were at least 10 s intervals between pacing sequences. VF was defined as an orderless rhythm without detectable QRS complexes lasting longer than 30 s. Normal SR was restituted by the transcutaneous defibrillation using pads placed in the conventional position sternum - apex (TEC-5521, Nihon Kohden, Japan). Biphasic shocks with rising energy (100-150-200-270 J) were delivered as needed. Another PVS procedure was performed at least 10 minutes after SR restoration in a previous one.

### Protocol

EP study began at minimum of 60 minutes after connecting ECMO. After the completion of 3 PVS procedures at the baseline (normothermia, NT), the cooling was started and other 3 PVS procedures were performed after reaching the core temperature of 32°C in pulmonary artery (hypothermia, HT). Finally, 3 PVS procedures were carried out after 60 minutes of stable hypothermia (HT60), Figure 2A.

### Statistics

Data are presented as median (interquartile range). Each animal was used as its own control and nonparametric pair Friedman test with Dunn’s multiple comparison post test were performed to assess the differences. The linear regression of the dependence of QTc interval on body temperature was performed after passing D’Agostino & Pearson omnibus normality test. The comparison of the VF inducibility was performed by Fisher’s exact test. The *P* values < 0.05 were considered significant. Statistical analysis and graphs were performed using Prism 5.0 (GraphPad, La Jolla, CA, USA).

## Results

**Figure 3:**
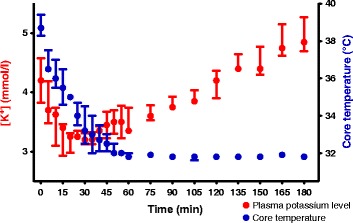
**The time dependence of the plasma potassium level and body temperature after the start of the cooling (time = 0).** Data are expressed as medians with interquartile ranges.

**Figure 4:**
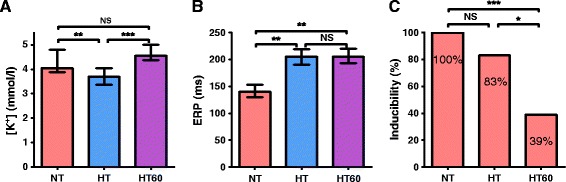
**Changes in potassium plasma level (A), effective refractory period (B) and inducibility of ventricular fibrillation (C).** Data are expressed as medians with interquartile ranges. NT indicates normothermia; HT, after reaching the core temperature of 32°C; HT60, after 60 minutes of stable hypothermia; NS, statistically non-significant; *P < 0.01; **P < 0.001; ***P < 0.0001.

**Figure 5:**
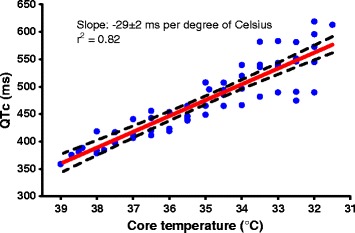
**Dependence of QT on body temperature with linear regression.** Error bars show 95% of confidence interval.

Overall, we used 7 animals (female, 4–5 months old, mean weight 51 ± 2 kg). One of them was excluded from analysis because of frequently reoccurring supraventricular tachyarrhythmias at the baseline. Providing ECMO cooling, the core body temperature dropped from normothermia of 38.7 (38.2; 39.8)°C to 32°C during 54 (39; 59) minutes and was further maintained constant at 31.9 (31.8; 32.0)°C, Figure 3. The potassium plasma level decreased along with the body temperature decline from basal 4.1 (3.9; 4.8) mmol/L to 3.7 (3.4; 4.1) mmol/L (*P* < 0.01) when reaching HT. In 3 of 6 animals (50%) dropped plasmatic potassium below 3.5 mmol/L, but none of them reached severe hypokalemia (<3.0 mmol/L). Subsequently, during maintaining of HT the potassium plasma level increased spontaneously again and at HT60 was similar to baseline level (4.6 (4.4; 5.0) mmol/L, *P* = NS, Figure 4A). Heart rate decreased from 148 (123; 161) at baseline to 85 (82; 100) beats per minute at HT (*P* < 0.001) and remained similar at HT60 (93 (81; 117) beats per minute). The QTc interval was significantly inversely related to the body temperature (−29 ± 2 ms per degree of Celsius, r^2^ = 0.82, Figure 5) and increased from basal 376 (362; 395) ms to 570 (545; 599) ms at HT. ERP adjusted to S1-S1 cycle length of 350 ms increased from 140 (130; 150) ms at NT to 206 (190; 220) ms when reaching HT (*P* < 0.001) and remained constant at HT60 (206 (193; 220) ms, Figure 4B). Through all the experiments, no spontaneously originating or provoked torsades de pointes (TdP) or heart conduction system disorders were observed. The inducibility of VF at normothermia was 100% (18/18 PVS), 83% (15/18 PVS) when reaching HT, *P* = NS, and 39% (7/18 PVS) at HT60, *P* < 0.0001, Table 1, Figure 4C.

**Table 1:** **Characteristics of ventricular fibrillation induction**

	**NT**		**HT**		**HT60**	
**Animal #**	**Induction parameter (ms)**	**Inducibility of VF**	**Induction parameter (ms)**	**Inducibility of VF**	**Induction parameter (ms)**	**Inducibility of VF**
1	400/200/140/110/100	3/3	400/220/130	3/3	400/270/170/160 (ARP)	0/3
2	350/150/110	3/3	350/200/170/160 (ARP)	0/3	350/220/140/130	3/3
3	300/150/90/80	3/3	350/240/140/130	3/3	350/230/160/150 (ARP)	0/3
4	350/140/60	3/3	400/250/180/170	3/3	400/240/180/170/160 (ARP)	0/3
5	350/150/140	3/3	350/220/180	3/3	400/230/220	1/3
6	300/120/60	3/3	400/230/160/150	3/3	400/230/200/190	3/3
Total		100% (18/18)		83% (15/18) NS		39% (7/18)***

Induction parameters represent minimal cycle lengths of basic stimuli and extrastimuli. In each animal and condition was EP protocol made three times. NT indicates normothermia; HT, after reaching the core temperature of 32°C; HT60, after 60 minutes of stable hypothermia. ARP indicates the achievement of absolute refractory period; NS, statistically non-significant; ***P = 0.0001.

## Discussion

In our experimental study we have proven that MTH is safe in terms of inducibility of malignant ventricular tachyarrhythmias in healthy pig hearts. Despite our hypothesis, the inducibility of VF was not increased both during the spontaneous transient decline of plasmatic potassium level and concurrent significant prolongation of QTc interval. Moreover, inducibility of VF was even significantly lower after normalization of potassium plasma level. Our data show that the potassium plasma level declines during the cooling phase, while during maintenance of MTH potassium plasma level rises spontaneously and ultimately returns to the baseline value.

Regarding dependence of potassium plasma level on body temperature, our data are concordant to clinical trials by Miryozev et al. [25], Soeholm and Kirkegaard [24]. Nevertheless, none of the animals in our experiment reached severe spontaneous hypokalemia (<3.0 mmol/L) which is supposed to be associated with a higher incidence of VT during cooling phase by the clinical trials. The exact mechanism of potassium plasma level changes remains not fully elucidated. In our study we can exclude the possible effects of drugs such as insulin, cathecholamines or diuretics, as well as significant ischemic damage of tissues or acid–base changes. Except for the suggested temperature related potassium reversible intra and extra cellular redistribution [31], Polderman et al. [32,33] documented hypothermia-induced polyuria caused by transient tubular dysfunction during the cooling phase which disappeared after maintaining MTH and rewarming. Based on our data, despite hypokalemia as a potential physiological phenomenon, maintaining the normokalemia during MTH contributed to electric stabilization of the myocardium. In accordance with other authors [25,27] we assume that close monitoring of potassium plasma level and its timely supplementation during the cooling phase could help decrease the risk of malignant ventricular arrhythmias.

As observed, the prolongation of QTc interval during MTH had not an adverse effect on arrhythmogenesis of ventricular arrhythmias. The QTc interval was inversely related to the body temperature and was independent of potassium plasma changes. Hypothermia related prolongation of QTc interval and decrease of heart rate were previously described in beagle dogs [34] and also in newborns [35]. According to our data the slope of the curve corresponds to the results in newborns well (-29 ms vs. -21 ms per degree of Celsius). Until recently, the occurrence of the long QT interval during MTH was considered to be a negative prognostic factor in terms of higher incidence of TdP or VF [27,36]. However, recent observational studies and meta-analysis did not prove this assumption [37-40]. Furthermore, as reported by Nishiyama et al. in case reports of congenital long QT syndrome [41], the induction of MTH after cardiac arrest did not have a proarrhythmic effect. Despite prolongation of QTc interval to extreme values during MTH, there were no recurrences of TdP documented. Similarly, in our experiments we have not detected any spontaneous or induced TdP taking into account the limitation in the use of healthy pig hearts.

Decrease in heart rate during MTH might be affected by decreased sympathetic activity as reported by Schwarzl et al. [42], which could also contributed to decreased VF inducibility. According to our previously published data, significant prolongation of ERP was observed as a sign of decreased sympathetic activity after renal denervation [43]. Nevertheless, considering comparable heart rate and ERP at HT and HT60 we can suppose that the tone of autonomic nerve system remained unchanged and could not play a major role in a significant decrease of VF inducibility at HT60.

The influence of profound reduction of myocardial temperature on VF was previously studied in more detail by Chorro et al. [44] in rabbit hearts using high-resolution epicardial mapping and transmural recordings of ventricular activation. They described the antiarrhythmic effect of deep hypothermia (<20°C) by exponential decay of VF dominant frequency, reduction in conduction velocity and subsequently single wave front extinction on activation maps. More recently, Harada et al. [45] proved antiarrhythmic effect of MTH in rabbit hearts. Although both MTH and deep hypothermia caused significant prolongation of action potential duration and significant reduction of conduction velocity, the duration of induced ventricular arrhythmias was significantly lower only in MTH. As they observed by high-resolution optical potential mapping, MTH causes a modification of arrhythmia’s spiral wave dynamics (annihilation or exit from the anatomical boundaries) leading to the increase of the chance of arrhythmia self-termination. This corresponds to the results of our study on the pig whole body model.

This study has several translational aspects related to post-resuscitation care. Frequent monitoring and adequate substitution of plasmatic potassium seems to be very important especially during the cooling phase to prevent malignant ventricular arrhythmias. On the other hand, close monitoring of QTc interval and premature termination of MTH due to long QTc appears not to be necessary. There is also potential in treating of incessant ventricular arrhythmias resistant to treatment when MTH could contribute to stabilization of the heart rhythm.

Our study was limited by using healthy animals not considering ischemic-reperfusion injury of the myocardium during the acute myocardial infarction or other predisposition for malignant ventricular arrhythmias. Thus, the translation of the results to humans should be interpreted with caution despite the fact, that our biomodel was represented by a breed repeatedly validated for simulation of human cardiac arrest and resuscitation [46,47]. The number of experimental animals tested is low, however multiple pairwise comparisons in individual animals counterbalances this limitation. The speed of cooling comparing ECMO cardiopulmonary resuscitation protocols [48] was considerably lower. Unlike them, we used a half ECMO flow (40 mL/kg/min) to prevent a hyperkinetic circulation in respect to spontaneous hemodynamics and also the priming the ECMO circuit with the precooled saline could not be applied before. Further, no other ions such as calcium or magnesium have been followed while they might play a significant role. We also did not analyze diuresis and ion excretion in urine. In present study, no potential mapping technics were used to describe exact mechanisms leading to antiarrhythmic effect of MTH preferring the complexity of the whole body biomodel than open-chest or isolated heart techniques.

## Conclusions

The induction of MTH in a swine model is safe with respect to the inducibility of malignant ventricular arrhythmias. Potassium plasma level decline during the cooling phase does not impair VF threshold and after achieving spontaneous normokalemia there is a significant effect on the reduction of VF inducibility. Prolongation of QT interval during MTH appears to be a physiological phenomenon reflecting the conduction changes and does not impair the inducibility of VF.

## Key messages

- MTH does not increase the inducibility of VF in a pig whole body model.
- Despite prolongation of QT interval, prolonged MTH seems to have an antiarrhythmic effect.

## Abbreviations

ARP: Absolute refractory period
ECG: Electrocardiogram
ECMO: Extracorporeal membrane oxygenation
EP: Electrophysiological
ERP: Effective refractory period
EtCO_2_: End-tidal carbon dioxide
HT: Hypothermia of 32°C
HT60: 60 minutes of stable hypothermia 32°C
MTH: Mild therapeutic hypothermia
NT: Normothermia
pCO_2_: Partial pressure of carbon dioxide
pO_2_: Partial pressure of oxygen
PVS: Programmed ventricular stimulation
RV: Right ventricle
SpO_2_: Peripheral capillary oxygen saturation
SvO_2_: Mixed venous oxygen blood saturation
TdP: Torsades de pointes
VF: Ventricular fibrillation
